# The measurement of impairment due to eating disorder psychopathology

**DOI:** 10.1016/j.brat.2008.06.012

**Published:** 2008-10

**Authors:** Kristin Bohn, Helen A. Doll, Zafra Cooper, Marianne O'Connor, Robert L. Palmer, Christopher G. Fairburn

**Affiliations:** a Oxford University Department of Psychiatry, Warneford Hospital, Oxford OX3 7JX, UK; b University Department of Health Sciences, Brandon Unit, Leicester General Hospital, Leicester LE5 4PW, UK

**Keywords:** Anorexia nervosa, Bulimia nervosa, Eating disorder, Impairment, Clinical significance, Diagnosis

## Abstract

Eating disorders have a profound and highly specific impact on psychosocial functioning. The aim of this research was to develop a measure of such secondary impairment. A 16-item, self-report instrument was developed, the Clinical Impairment Assessment (CIA), which was designed to measure such impairment overall and in three specific domains (personal, cognitive, social). The psychometric properties of the instrument were evaluated using data collected in the context of a transdiagnostic treatment trial. The findings consistently supported the utility of the instrument with the CIA being shown to have high levels of internal consistency, construct and discriminant validity, test–retest reliability, and sensitivity to change. The CIA should be of value to clinicians when assessing patients with eating disorders and their response to treatment. It should also help inform epidemiological research.

## Introduction

The assessment of psychopathology requires not only an evaluation of the nature and severity of particular features, but also an assessment of the impact of these features on the person's psychosocial and physical functioning. This is important for at least two reasons: first, it is often impairment that leads people to seek help and a goal of treatment should therefore be to reduce it; and second, the presence of clinically significant impairment is required to make a diagnosis of a mental disorder (American Psychiatric Association, 1994). Despite this, measures of psychopathology have tended to focus on the psychopathology itself and not the impairment that it causes. Recently this tendency has been countered to an extent with the increasing use of measures of “health-related quality of life” to supplement the assessment of symptoms. Although valuable, these generic measures, developed originally to assess the impact of physical illnesses on everyday functioning (Ware, Kosinski, & Keller, 1996; Ware, Snow, Kosinski, & Reese, 1993; WHOQOL Group, 1998), may miss important sources of impairment that are peculiar to psychopathology and to the characteristics of particular psychiatric disorders.

Eating disorders are a case in point for they have profound and specific effects on psychosocial functioning. For example, these patients' over-evaluation of shape and weight and its expressions, the so-called “core psychopathology” (Fairburn, 2008), has a marked effect on their ability to be with others and to form intimate personal relationships. Similarly, their concerns about eating, and its expressions, prevent them from eating healthily, affecting their mood, cognitive function and family relationships. Secondary effects of this type can be extremely disabling yet are likely to be missed by generic measures of health-related quality of life (Doll, Petersen, & Stewart-Brown, 2005). For this reason disorder-specific measures are required.

Four eating disorder-specific measures of health-related quality of life have recently been developed (Abraham, Brown, Boyd, Luscombe, & Russell, 2006; Adair et al., 2007; Engel et al., 2006; Las Hayas et al., 2006). However, none of the four measures is entirely satisfactory as a measure of impairment secondary to the whole range of eating disorder psychopathology. The main problems are as follows. First, three of the instruments confound the measurement of eating disorder psychopathology with the assessment of impairment and fail to ensure that the impairment assessed is secondary to eating disorder psychopathology (Abraham et al., 2006; Adair et al., 2007; Las Hayas et al., 2006). Second, three instruments omit to assess the impact of the patients' extreme concerns about their shape (Abraham et al., 2006; Engel et al., 2006; Las Hayas et al., 2006) and as a result are likely to underestimate the extent of the secondary impairment. Third, sensitivity to change was only examined in one of the four studies (Abraham et al., 2006). Fourth, none of the instruments have been validated against independent assessments of the extent of secondary impairment and none has been evaluated in terms of its ability to predict case status.

The aim of the present study was to develop a clinically useful measure of the psychosocial impairment that results from eating disorder features and to test its reliability, validity, sensitivity to change and ability to predict case status.

## Methods

### Development of the CIA

It was decided a priori that the instrument should have certain properties to ensure it would function as an easy-to-use measure of psychosocial impairment secondary to eating disorder features. First, it needed to be a self-report questionnaire. Second, it needed to be compatible with a current-state measure of eating disorder features so that together the two instruments would provide an assessment of psychopathology and its resulting impairment. The Eating Disorder Examination Questionnaire (EDE-Q) (Fairburn & Beglin, 1994) was chosen as the measure of eating disorder psychopathology as it is widely used and has been extensively validated (Peterson & Mitchell, 2005). Thus the new measure of impairment, named the Clinical Impairment Assessment (CIA), was designed to have the same Likert-style response format as the EDE-Q and the same time frame (covering the previous 28 days) so that respondents could easily move from completing one instrument to the completion of the other. Third, it was decided that the CIA should be relatively brief so that the two instruments could be completed together in a short period of time (approximately 10 min). Fourth, to maximise clinical utility, it was decided that the instrument should generate a single, readily-calculated, overall score indicative of the severity of secondary impairment, although the possibility of generating domain-specific scores was to be explored. Lastly, it was decided to focus exclusively on secondary psychosocial impairment rather than the physical effects of eating disorder psychopathology as the subjective physical consequences of an eating disorder (e.g., weakness, feeling faint or cold, palpitations, muscle twitches and spasms) are mostly non-specific in character and difficult for the individual to ascribe to their way of eating.

Two main considerations governed decision-making regarding the content of the CIA. First, it needed to assess the influence of all the main elements of eating disorder psychopathology on a person's functioning. Hence it was decided that the CIA should open with the following stem question: “Over the past month, to what extent have your eating habits, exercising or feelings about your eating, shape or weight affected…”. We decided not to include purging in the stem question as “eating habits” includes vomiting and laxative misuse in most patients' minds and, having just completed the EDE-Q, participants will have these forms of behaviour in their mind. Second, it was decided that the CIA should ask about the main aspects of life that are affected by eating disorder psychopathology. These were identified by KB, ZC, CGF and RLP on the basis of their clinical experience, the content of generic measures of health-related quality of life, and the responses of eating disorder patients to exploratory interviews focused on the presence and nature of any secondary psychosocial impairment that they were experiencing (Bohn, 2006). Examples of impairment within the identified domains of life were specified resulting in the eventual development of a 22-item instrument with 7 items directed at effects on mood and self-perception, 4 at effects on cognitive functioning, 7 at impairment of interpersonal functioning, and 4 at effects on work performance. Each item was rated on a 4-point Likert scale, where 0 (“Not at all”) was equivalent to no impairment and 3 (“A lot”) to severe impairment. The total score (‘global CIA score’) was designed to provide an overall index of severity of current secondary psychosocial impairment. This preliminary version of the CIA was evaluated using data collected in the context of a treatment trial.

### Assessment of the psychometric properties of the CIA

#### Participants

CIA data were collected from 123 of 170 patients who were participating in a transdiagnostic cognitive behaviour therapy trial based in two eating disorder clinics in the UK (Oxford and Leicester) (Fairburn et al., in press). Both clinics provide the only secondary adult eating disorder service for the locality. Patients were included if they met the following criteria: aged 18–65 years, judged to have an eating disorder of clinical severity by one of three senior specialists in the field (ZC, CGF or RLP), and had a body mass index between 16.0 and 39.9. The CIA data of patients who were diagnosed as suffering from a severe co-existing clinical depression were excluded as some of their impairment might have been secondary to the clinical depression rather than their eating disorder features. Details of how this judgement was made are provided elsewhere (Fairburn, Cooper, & Waller, 2008).

The CIA was introduced after 120 of the 170 patients had started treatment with the consequence that pre-treatment CIA data were collected on only 50 of the patients. A further 73 patients contributed data between the end of treatment and end of their post-treatment follow-up. Of these 123 patients, 27 (22.0%) completed the CIA once, 44 (34.9%) twice, 30 (24.4%) three times, 17 (13.8%) four times, and 5 (4.1%) five times, with there being 298 CIA ratings in total over all patients and assessment points. The baseline DSM-IV diagnoses of the 123 patients were as follows: anorexia nervosa – 8 (6.5%); bulimia nervosa – 48 (39.0%); eating disorder NOS – 67 (54.5%). Full details of the complete sample and its response to treatment are provided elsewhere (Fairburn et al., in press).

#### Assessments

Each patient underwent a research assessment at the beginning and end of their treatment, and at 20, 40, 60, 104 and 208 weeks post-treatment. At each point they completed the EDE-Q and, immediately afterwards (from the time of its introduction), the CIA. In addition, a trained research assistant administered the Eating Disorder Examination (EDE) interview (Fairburn & Cooper, 1993) together with an investigator-based interview designed to identify secondary functional impairment (Bohn, 2006). In Oxford, ZC and CGF also independently assessed (using an unstructured clinical interview) the extent to which the patient's eating disorder features had been interfering with their psychosocial functioning over the previous 28 days and rated this on a seven-point severity scale (0–6, with 0 being equal to no secondary impairment and 6 being equal to severe impairment). Finally CGF determined whether the patient should be viewed as suffering from an eating disorder of clinical severity. This decision was based upon ratings on the EDE and the investigator-based impairment interviews, and was made blind to the patient's identity, point of follow-up and responses to the EDE-Q and CIA. A second CIA was administered to a subset of 43 participants three days after they had completed an initial one. This addressed the same 28-day period.

#### Statistical methods

Unless otherwise stated, the analyses used the data from the first CIA completed by each participant so as not to violate the assumption of statistical independence. When data collected across multiple assessments were used to increase statistical power, a variety of multilevel models, with a random effect for subject, were fitted to allow for correlation between repeated assessments and to assess the impact of statistical non-independence (Singer & Willett, 2003). Statistical significance was taken throughout at two-tailed *p* < 0.05.

##### Internal consistency and dimensionality

Cronbach's alpha and item-total correlations were used to assess internal consistency. A principal components factor analysis with varimax (constraining the factors to be independent) and oblimin (allowing the factors to be associated) rotations was used to assess dimensionality. In addition, Item Response Theory (IRT) with the fitting of a one-parameter Rasch model was used to explore the unidimensionality of the questionnaire and any identified domains, and to assess the performance of each item (Andrich, 1988; Avlund, Kreiner, & Schultz-Larsen, 1993; Hambleton & Jones, 1993). A Rasch model is equivalent to a test of the theoretical construct validity and adequacy of a scale. It assumes that as a person's disability or symptoms increase, the probability of a maximum score on each item increases.

##### Test–retest reliability

An intra-class correlation coefficient (ICC) was calculated between the global CIA scores at the two time points. In addition, a paired samples *t*-test was used to examine any difference between the pairs of scores.

##### Construct validity

Two tests of construct validity were performed. First, on the assumption that there would be a positive association between the severity of eating disorder features and the extent of psychosocial impairment secondary to them, mean global CIA scores were compared with the global EDE-Q score using Spearman's correlation coefficient. The second and stronger test of construct validity involved comparing mean global CIA scores with the corresponding clinicians' impairment ratings. This comparison was performed at the first assessment conducted on each patient, over all time points and at each individual time point to assess whether the agreement was independent of point of assessment. In addition, over all assessments, a one-way ANOVA was performed to examine the relationship between the global CIA score and the clinicians' impairment ratings (with clinicians' ratings of 5 and 6 being combined since a rating of 6 was made only once out of the 142 ratings). Post-hoc tests were performed using Tukey's B procedure. Finally, a test of linearity was conducted. Multilevel models were fitted to allow for correlation between repeated measures.

##### Discriminant validity

The discriminant validity of the CIA was first assessed using an independent samples *t*-test comparing the global CIA scores of participants who were, and were not, classed as having an eating disorder of clinical severity. The analysis was repeated at each assessment and over all assessments, with multilevel models being fitted to allow for correlation between repeated measures. Second, the performance of the CIA at predicting patients' case status was tested using a Receiver Operating Curve (ROC) signal detection analysis. This involved plotting sensitivity against 1 minus specificity for each possible cut-off point on the global CIA score, and on each domain score, thereby obtaining a full profile of the instrument's performance (Charman et al., 2007; Hanley & McNeil, 1982).

##### Sensitivity to change

Sensitivity to change was tested in two ways: first, pre- and post-treatment CIA data were compared using a paired samples *t*-test; and second, changes in global CIA scores from pre- to post-treatment were compared with change in psychosocial impairment as rated by the clinicians using Spearman's correlation coefficient.

## Results

### Internal consistency and dimensionality

#### The 22-item preliminary version of the CIA

The internal consistency of the 22-item CIA at first completion was high (Cronbach's alpha = 0.97). All items correlated positively with the global CIA score, with the item-total correlations ranging from 0.59 to 0.85. On IRT analysis, the 22-item questionnaire was significantly non-unidimensional (*X*^2^ = 61.7, df = 44, *p* = 0.04).

#### The 16-item CIA

Since the 22-item CIA was not unidimensional and contained five items that either had a notable floor effect, significantly misfit, or whose responses were inconsistent with the underlying construct (full details available from the authors), these items were excluded leaving a 17-item instrument. On principal components factor analysis 2 of the 17 items did not load clearly on any one domain. Since one of these items was judged to be of particular clinical relevance and had the highest initial item-total correlation (0.85), it was retained.

On principal components factor analysis, the resulting 16-item instrument (the CIA) fell into two components explaining 71% of the variance. The larger component consisted of one set of items with high loadings (>0.86) and one set with low loadings (between 0.41 and 0.59). Forcing a three component solution generated three factors (see Table 1) which explained 77% of the variance. The three factors might best be described as personal, social and cognitive impairment, respectively. The varimax and oblimin solutions generated similar factors. On IRT analysis, the instrument was unidimensional (*X*^2^ = 32.6, df = 32, *p* = 0.44), as was each of the three individual domains, making it appropriate to create both global and domain-specific scores. The Cronbach's alpha for the 16-item instrument was 0.97. The overall mean (SD) score was 20.1 (13.4), with a range of 0–47 (possible maximum score 48).

### Test–retest reliability

The mean (SD) scores of the 16-item CIA at times 1 and 2 were 10.56 (7.58) and 9.02 (8.18), respectively. While the small reduction in score (0.20 of a standard deviation) was statistically significant (*p* = 0.022), the intra-class correlation coefficient was high at 0.86 (95% CI 0.75–0.92; *p* < 0.001) indicating acceptable overall test–retest reliability.

### Construct validity

Significant positive correlations were found between global scores on the 16-item CIA and both, scores on the EDE-Q (*r*_s_ = 0.89, *p* < 0.001) and the clinicians' impairment ratings (*r*_s_ = 0.68, *p* < 0.001) (see Table 2). These relationships were evident at each time point (see Table 2) and remained (both *p* < 0.001) on fitting multilevel models.

**Fig. 1:**
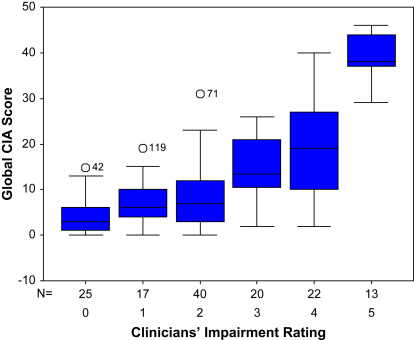
Box plot of mean global CIA scores for the five levels of clinician-rated impairment.

Fig. 1 shows a box plot diagram of the global CIA scores for each of the different severity ratings made by the clinicians. There was a significant deviation from linearity (*F*(4) = 11.9, *p* < 0.001), reflecting the fact that three homogeneous groups were found on post-hoc testing: those who received ratings of 0, 1 and 2 (little or no impairment); those with ratings of 3 or 4 (moderate impairment); and those with a rating of 5 or 6 (severe impairment).

### Discriminant validity

The CIA global and domain scores of the 37 participants who (at their first CIA assessment) were judged to no longer have an eating disorder were significantly lower than those of the 33 participants who were judged to still be a “case” (global CIA score: 7.86 (6.83) vs 27.64 (12.44); *t* = 8.36, df = 68, *p* < 0.001) with similar scores being observed across all 137 pairs of ratings. On fitting multilevel models to allow for correlation between repeated assessment, significant effects of eating disorder status were observed for the global and domain scores (all *p* < 0.001).

**Fig. 2:**
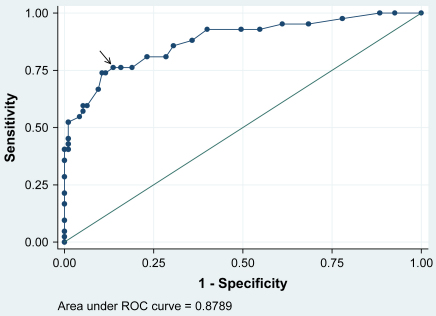
ROC curve for the prediction of clinician-rated eating disorder status from the global CIA score, with the arrow indicating the best cut-point (*N* = 137).

ROC analysis showed that the global CIA score was better able to predict case status (area under curve = 0.88) than the three individual domains (personal, social, and cognitive domains 0.87, 0.85, and 0.80, respectively; with the cognitive domain performing significantly less well (*p* = 0.0014)). The best cut-point was a global CIA score of 16, which had a sensitivity of 76% and specificity of 86% (Fig. 2).

### Sensitivity to change

Participants' mean (SD) global CIA score dropped significantly from 31.2 (9.88) at the start of treatment to 8.22 (10.7) at the end (*t* = 9.76, df = 22, *p* < 0.001). Similar changes were observed in each domain score (all *p* < 0.001). In addition, there were significant positive correlations between change in global CIA score and change in the clinicians' impairment ratings (global score *r*_s_ = 0.86, *p* = 0.013).

## Discussion

Eating disorders have a profoundly negative impact on patients' lives, yet to date there is no satisfactory measure of this impairment. Such a measure would be of great value in the clinical assessment of patients and their response to treatment. It would also help inform epidemiological studies of the burden imposed by eating disorder psychopathology.

The aim of this research was to develop a clinically useful measure of the psychosocial impairment secondary to eating disorder features and to test its psychometric properties. To this end the 16-item, self-report CIA was developed and its reliability, validity, sensitivity to change and ability to predict eating disorder case status were evaluated using data collected in the context of a treatment trial.

It was found that the internal consistency of the preliminary 22-item instrument was high and that all items correlated positively with the global score. However, IRT analysis showed that the questionnaire was significantly non-unidimensional and that six items either had a notable floor effect, significantly misfit or were inconsistent with the underlying construct. These items were therefore excluded. The resulting 16-item instrument (the CIA) was found to be unidimensional and showed high internal consistency. Principal component analysis suggested the existence of three factors (named ‘personal’, ‘social’, and ‘cognitive’), explaining 77% of the variance. All three domains were also found to be unidimensional, making it possible to use both global and domain-specific scores. Overall the findings suggest that the CIA measures one construct. Given the content of the instrument and the fact that its scores correlate closely with clinicians' ratings of secondary psychosocial impairment, it is highly likely that it is measuring psychosocial impairment secondary to the presence of eating disorder features.

The test–retest reliability of the CIA was examined by administering the CIA twice within three days to participants with varying degrees of eating disorder psychopathology. It was found that there was a statistically significant positive correlation between the CIA scores at the two time points. The scores were on average slightly lower on the second occasion but the difference was small. Overall, the findings suggest that the CIA has satisfactory test–retest reliability.

Two tests of the construct validity of the CIA were conducted. The first was an indirect one and it involved comparing scores on the EDE-Q and the CIA. A statistically significant positive correlation was found indicating that higher levels of eating disorder psychopathology were associated with higher levels of secondary psychosocial impairment. The second test involved comparing scores on the CIA with ratings of secondary psychosocial impairment made by expert clinicians. Again there was a strong positive correlation between the two.

The discriminant validity of the CIA was tested by comparing the global and domain-specific CIA scores of patients with an eating disorder with those of patients who were judged no longer to have one. It was found that the scores of the two groups differed significantly. A ROC analysis revealed that a cut-point of 16 on the CIA best predicted eating disorder case status, with a sensitivity of 76% and a specificity of 86%.

Lastly, sensitivity to change was tested. It was found that there was a significant decrease in patients' CIA global and domain scores following cognitive behaviour therapy, and that there were significant positive correlations between change in clinician-rated secondary impairment and change in the global CIA score.

Certain points about this work are of note. First, this new instrument, the CIA, addresses impairment due to all the main elements of eating disorder psychopathology including concerns about shape. None of the other measures explicitly assesses the effect of such concerns despite the fact that they are central to the “core psychopathology” of the eating disorders (Fairburn, 2008). In this study, patients are likely to have had all the main features of their eating disorder at the forefront of their mind when completing the CIA, as they were asked to complete the EDE-Q, a measure of all aspects of eating disorder psychopathology, immediately before the CIA. A second strength of this research is that many aspects of the performance of the CIA were tested including sensitivity to change and ability to predict case status. Equivalent data are not available for existing measures of eating disorder-specific health-related quality of life. Third, the sample used is likely to have been representative of many other outpatient samples of adults with an eating disorder, given its transdiagnostic composition and the two catchment area-based sampling frames. Fourth, the participants were assessed both before and after treatment and over an extended period of follow-up. Therefore CIA data were available on people with the full range of severity of eating disorder psychopathology. This was important since we wanted to test the CIA's performance across its entire scoring range. Lastly, the ability of the CIA to assess secondary psychosocial impairment was validated against a simultaneous but independent assessment of impairment made by expert clinicians, the best available validator.

The main limitation of the study was the relatively small sample size, especially given the need to employ subsets of the data for the various analyses. Despite this, significant associations were found, all of which support the validity and utility of the CIA. Another limitation is that the sample contained few patients with anorexia nervosa. More data are needed on this subgroup of patients. It might be thought that a limitation of the study of discriminant validity was the fact that no CIA data on healthy controls were used: instead, discriminant validity was tested by comparing patients with a clinical eating disorder with patients who were judged to no longer to have one. In fact this was a particularly stringent test since the latter participants were likely to have residual levels of eating disorder psychopathology making discrimination more difficult. Nevertheless it would be of value to have normative data on the CIA to ascertain the degree of impairment that is associated with the level of eating disorder features found in the general population. As always, it is important to stress that the findings require replication, preferably using larger samples and ones differing in case composition, age and ethnicity. Finally, it should be recalled that the CIA does not attempt to assess secondary physical impairment. This is because we do not think it is possible for patients to reliably identify those aspects of their physical functioning that are being impaired exclusively as a result of their eating disorder. We recommend that any assessment of secondary physical impairment should be made by a physician alongside the administration of the CIA.

In conclusion, this paper has described the development and performance of a brief self-report measure of the nature and severity of the psychosocial impairment that arises from eating disorder psychopathology. The findings support the validity and utility of the instrument.

## Figures and Tables

**Table 1:** Component matrix showing rotated (varimax) loadings of each item on the three extracted components of the final 16-item CIA questionnaire

	Component		
	1	2	3
	Personal impairment	Social impairment	Cognitive impairment
Eigenvalue	9.52	1.12	0.93

*Item*			
Over the past month, to what extent have your eating habits, exercising, or feelings about your eating, shape or weight…			
made you feel ashamed of yourself	0.86		
made you feel guilty	0.84		
made you feel critical of yourself	0.80		
made you feel a failure	0.77		
made you upset	0.75		
made you worry	0.71		
interfered with meals with family or friends		0.80	
made it difficult to eat out with others		0.80	
interfered with you doing things you used to enjoy		0.71	
stopped you going out with others		0.63	
interfered with your relationship with others		0.55	
made you absent-minded			0.85
made you forgetful			0.82
affected your ability to make everyday decisions			0.68
affected your performance at work (if applicable)			0.63
made it difficult to concentrate			0.45

Mean (SD) score	10.2 (6.05)	5.36 (4.58)	4.51 (4.01)
Median (range)	11 (0–18)	5 (0–15)	4 (0–15)

*p*-Value for unidimensionality	0.46	0.97	0.65

**Table 2:** Mean (SD) global CIA score and clinicians' impairment ratings at each assessment and overall, with Spearman's correlation coefficients

Assessment	*N*	Global CIA score (0–48)	Clinicians' impairment rating (0–6)	*r*_s_ (*p*)
Beginning of treatment	23	32.5 (11.1)	4.39 (0.72)	0.692 (<0.001)***
End of treatment	27	7.85 (5.43)	2.11 (1.16)	0.322 (0.102)
20-Week follow-up	22	11.0 (8.87)	1.82 (1.26)	0.454 (0.034)*
40-Week follow-up	25	8.44 (7.08)	1.68 (1.35)	0.471 (0.017)*
60-Week follow-up	18	10.2 (9.10)	1.50 (1.38)	0.590 (0.010)*
2-Year follow-up	16	8.56 (7.50)	1.69 (1.35)	0.633 (0.008)**
3-Year follow-up	6	14.0 (12.1)	2.67 (1.75)	0.924 (0.008)**

Total	137	13.3 (12.1)	2.26 (1.56)	0.676 (<0.001)***

*Significant at 0.05 level. **Significant at 0.01 level. ***Significant at 0.001 level.
